# Exploring the Swimming and Water Safety Behaviour Among Indian and Vietnamese Adults in Australia

**DOI:** 10.1002/hpja.70163

**Published:** 2026-03-02

**Authors:** Lian Low, Hannah L. M. Graefe, Stacey M. Willcox‐Pidgeon, Lisa M. Barnett

**Affiliations:** ^1^ School of Health and Social Development, Faculty of Health Deakin University Geelong Victoria Australia; ^2^ Life Saving Victoria Port Melbourne Victoria Australia; ^3^ Centre for Alcohol Policy Research La Trobe University Melbourne Victoria Australia; ^4^ Royal Life Saving Society Australia Sydney New South Wales Australia; ^5^ James Cook University College of Medicine and Dentistry Townville Queensland Australia; ^6^ School of Health and Social Development Institute for Physical Activity and Nutrition, Deakin University Geelong Victoria Australia

**Keywords:** drowning, swimming, water safety behaviour

## Abstract

**Issue Addressed:**

From 2011 to 2021, drowning caused over 2.5 million preventable deaths, with Asia carrying the highest burden of drowning deaths. India and Vietnam are among the top 10 birth countries of people who drown in Australia. This study aimed to understand how Asian adults living in Australia develop health literacy in relation to swimming ability and water safety and how these skills are implemented in different aquatic environments.

**Methods:**

Three men and nine women between 20 and 79 years old born in India and Vietnam and living in Victoria, Australia were recruited using convenience and snowball sampling. Semi‐structured online interviews were audio recorded, transcribed and thematically analysed using a deductive approach.

**Results:**

Three interrelated themes emerged. First, there were cultural differences in how participants related to aquatic environments in their birth countries compared to Australia and the impact this had on their health literacy. Secondly, family influence on perceptions of aquatic environments determined participants' perception of risk and safety in aquatic environments. The last theme related to the differences in access, affordability and availability of swimming and water safety education and how this impacted participants' swimming ability.

**Conclusions:**

A multisectoral approach is recommended involving councils, key multicultural organisations and community leaders to increase awareness of drowning prevention related health literacy within Asian communities and to implement targeted water safety education and swimming programmes.

**So What:**

Targeted education, engagement and co‐designing programmes with community leaders are needed to respond to the emerging trend of fatal drowning in Asian communities in Australia.

## 1 Introduction

Despite drowning being preventable, 2762 fatal drownings occurred in Australia between 2013 and 2023: with 34% involving people born overseas [1]. Notably, the top 10 countries of birth included India and Vietnam, where 26% had lived in Australia for 30+ years [1]. In the State of Victoria, between 2012 and 2022, people born overseas comprised 33% of drowning deaths. The mean length of time living in Australia for this group was 20 years [2], indicating this issue does not just impact new arrivals. In 2021, India and Vietnam were among the top five countries recording population increases within Australia, with India recording the highest numbers [3]. As such, India and Vietnam are among the priority culturally and linguistically diverse (CALD) populations for Australian drowning prevention.

The Australian Water Safety Strategy (AWSS) 2030 is the national strategy for drowning prevention, where CALD or multicultural communities are priority groups for drowning prevention [4]. In the AWSS 2030, multicultural communities are identified as migrants, international students and overseas visitors [4]. For the purpose of this study and for consistency of terminology, the term CALD is selected to include people born overseas, migrants, international students and overseas visitors. These groups have been identified as having lower swimming and water safety skills that could be attributed to social determinants of health such as cost and access to swimming and water safety programmes as well as social norms and cultural factors [4].

A review of high‐income countries reported that migrants were at increased drowning risk, due to less familiarity with their new geographical, social and cultural environment [5]. The review reported that adult migrants may be more vulnerable to drowning compared with children, due to the focus on child swimming and water safety education [5]. It has been recommended that cultural background, including attitudes and perceptions, is important to consider when implementing prevention strategies [5, 6].

There is a growing body of Australian literature related to drowning prevention in Asian communities in Australia, including Vietnamese and Chinese rock fisher behaviours and perceptions [7], South Asian Australian community's beachgoer safety knowledge and behaviour [8], and strategies to promote water safety knowledge among Chinese speakers in New South Wales [9]. However, there is a gap in understanding the swimming ability, water safety knowledge, attitudes and cultural beliefs of Indian and Vietnamese communities living in Australia. One report identified risk factors for drowning relating to Indian and Vietnamese communities, which included: lack of access to formal swimming lessons from the country of origin; gender and modesty as considerations; and the lack of water safety awareness in Australian aquatic environments [10].

Furthermore, there is limited knowledge in how CALD communities develop health literacy in relation to water safety and drowning prevention, and how the skills are implemented [11]. Sørensen et al. define integrated health literacy as a lifelong journey linked to knowledge, motivation and competence to access, understand, appraise and apply health information in everyday life [12]. While early definitions of health literacy focused on defining individual cognitive and social skills, there is increasing recognition of health literacy as multidimensional and context‐specific, taking into consideration the role of systems, and the interaction of individuals with health and community services in promoting and maintaining health [13, 14]. Thus, health literacy in the drowning prevention and water safety context refers to a set of skills including knowledge, attitudes and personal skills that influence decision making and behaviour [11].

Models of health literacy do not explicitly include cultural literacy [12]. Cultural literacy refers to the ability to recognise and use ‘collective beliefs, customs, world‐view and social identity relationships to interpret and act on (as well as produce) health information’ [15, p. 120]. Additionally, individuals from CALD communities appear less likely to engage in physical activity compared to people from non‐CALD backgrounds in Australia [16]. These factors can be partially explained under the banner of ‘physical literacy’ which relates to the lifelong holistic learning as defined by the Australian Physical Literacy Framework [17]. This framework identifies 30 elements across the four physical literacy domains—physical, psychological, social and cognitive. Each element has five stages of learning development to outline progression or regression in one's life course. Therefore, this framework can be used as a guide in how an individual understands the stages of their swimming development and knowledge acquisition related to water safety.

In summary, despite work undertaken in drowning prevention, Asian communities in Australia remain vulnerable and a priority area for drowning prevention. Therefore, this study aimed to investigate how Asian adults in Australia develop and implement their health literacy skills in relation to water safety and drowning prevention using the lenses of health, cultural and physical literacy.

## 2 Methods

### 2.1 Design

An exploratory, qualitative descriptive method allowed for in‐depth insight into human behaviour, motives and views while staying true to the participant's account [18]. The methods followed the Consolidated Criteria for Reporting Qualitative Research tool to improve rigour, comprehensiveness and credibility of the qualitative study [19] (Table S2).

### 2.2 Research Team and Positionality

The research team comprised four members: a genderqueer Public Health and Health Promotion Honours student (LL) with a Malaysian migrant background and three women without CALD backgrounds—one academic (LB) (> 15 years) and two industry researchers in drowning prevention (> 15 years [SW‐P] and > 10 years [HG]). Interviews were conducted by the lead researcher (LL), who disclosed the study's Honours context. To reduce power imbalances, questions were in plain English, participation was voluntary, and opt‐out was allowed. Bias was addressed through team reflection on positionality and privilege during analysis.

### 2.3 Ethics

This study has received Deakin University ethics approval (Ref number: 2024‐079).

### 2.4 Materials

A semi‐structured interview guide was developed to understand how health, cultural and physical literacy affect swimming and water safety practices (Table S3). Upon ethical approval, the interview guide was piloted with three overseas‐born Australia‐based postgraduate university students to test for clarity, identify ambiguity and assess if the timing and structure worked under the literacy theory frameworks. Through a reflective process with the research team, the pilot interviews resulted in minor amendments to some questions to ensure comprehension in plain English.

For health literacy, interview questions related to four competencies: access (ability to find information); understand (ability to comprehend accessed information); appraise (ability to interpret and evaluate accessed information); and apply (ability to communicate and use the accessed information to make an informed decision in maintaining and improving health) [12].

For cultural literacy, questions encompassed the antecedents of health literacy such as societal and environmental determinants (culture, language and societal systems), situational determinants (family and peer influences, physical environment), and personal determinants (gender, race, socio‐economic status, employment, income and literacy) [12]. For physical literacy, topics related to selected elements within the four Australian Physical Literacy Framework (the Framework) domains: physical, psychological, cognitive and social [17]. The physical domain includes the elements of ‘movement skills’ and ‘moving with equipment’, both selected as they relate to the development of swimming strokes and the use of boats. The psychological domain includes the elements of ‘self‐perception’, ‘motivation’, ‘confidence’ and ‘connection to place’ which relate to swimming ability, motivation to learn and the exploration of different aquatic environments. The cognitive domain includes the element of ‘safety and risk’, related to understanding of risks, risk management, identifying water safety guidelines when swimming or recreating at different aquatic environments. Finally, the social domain includes the element of ‘society and culture’ related to the appreciation of cultural values.

Photo elicitation was used to complement the interview questions to explore differences in participants' relationship to coastal and inland waterways in Australia, compared to their birth country. This technique aimed to evoke insights and memories from participants to enable further discussion [20]. When questions had visual prompts, the researcher shared images on their computer screen as a Microsoft PowerPoint presentation (Table S4).

### 2.5 Participants and Recruitment

Between June and July 2024, recruitment flyers were disseminated to councils, community organisations and multicultural networks in Greater Melbourne, and participants were recruited using a convenience and snowball sampling approach. Inclusion criteria were: adults (≥ 18 years); born in, and/or had family ties to Asia; resided in Victoria; and were able to provide informed consent and communicate in English. Participants were recruited on a first come, first served basis using a convenience and snowball sampling approach. A minimum of 12 participants were deemed appropriate for a heterogenous sample and to achieve maximum variation [21]. After the interview, participants were offered an AUD$25 supermarket e‐gift card as reimbursement for their time.

### 2.6 Data Collection

Prior to the interview, participants were sent a short survey via Qualtrics collecting basic demographic information and to determine their proficiency in English by checking their responses to the questions before commencing the interview. The semi‐structured one‐on‐one interviews conducted via an online platform (Zoom/Microsoft Teams) lasted 30–45 min, facilitated by the lead researcher, and were audio recorded and transcribed verbatim using Panopto software.

### 2.7 Analysis

Braun and Clarke's framework for reflexive thematic analysis was applied to the de‐identified verbatim transcriptions of the interview recordings. This involved: reviewing and confirming accuracy of the transcripts against audio recordings, familiarisation with the transcripts, and coding and reviewing codes and themes with the research team [22, 23]. Interview transcripts were shared with participants to check for the accuracy of the data as part of member checking prior to commencing analysis [24]. Participants were asked to provide feedback within 1 week, with no response in that time deemed approval of the transcript.

To ensure research rigour, the research team reviewed codes and themes during data analysis [25]. Two transcripts were initially coded independently by three members of the research team to ensure consistency of coding, with any disagreements resolved through discussion. The lead researcher then coded the remaining transcripts using NVivo V15 [26], while simultaneously generating a codebook to refine the definition of the eventuating 16 codes ensuring a systematic approach. Throughout the analysis, codes, themes and sub‐themes were reviewed and discussed among the research team [22, 23]. While the initial process involved the inductive coding of transcripts, the subsequent deductive coding definitions were derived and adapted from Sørensen et al.'s integrated health literacy framework [12] and the Australian Physical Literacy Framework [17] to incorporate the dimensions of health, physical and cultural literacies (Table S1).

## 3 Results

Nine women and three men aged 20–79 years participated. While study eligibility was for adults born in or with family ties to any Asian country, only two countries were captured in the sample, and participants were either born in India or Vietnam (Table 1). Years in Australia (YIA) ranged from 2 to 40 years. Five identified as non‐swimmers, five identified as swimmers and two were learning how to swim.

**TABLE 1:** Participant demographics.

ID	Postcode	Country of birth	Years in Australia	Which community/communities do you identify with? (e.g., Chinese, Indian,‐ Vietnamese, etc.)	Age	Gender	Languages spoken	How well do you speak and understand English?	Self‐perception of swimming ability (swimmer/non‐swimmer/learning to swim)
P1	3075	India	7	Indian	30–39	Woman or female	Hindi, Marathi, English, learning Sanskrit and German	Very well	Non‐swimmer
P2	3031	Vietnam	39	Vietnamese	60–69	Man or male	English and Vietnamese	Well	Non‐swimmer
P3	3023	Vietnam	34	Vietnamese	50–59	Woman or female	English and Vietnamese	Well	Swimmer
P4	3150	India	2	Indian	40–49	Woman or female	English, Hindi and Marathi	Very well	Non‐swimmer
P5	3031	Vietnam	36	Vietnamese	60–69	Woman or female	Vietnamese, English	Well	Swimmer
P6	3036	India	40	Indian	70–79	Woman or female	English, Hindi, Marathi and Konkani	Very well	Learning to swim
P7	3335	India	12	Indian	30–39	Woman or female	English and Hindi	Very well	Learning to swim
P8	3150	India	12	Indian	20–29	Man or male	English, Hindi and Marathi	Very well	Swimmer
P9	3183	India	11	Indian	30–39	Woman or female	English, Tamil and Hindi	Very well	Non‐swimmer
P10	3337	India	16	Indian	30–37	Man or male	English, Punjabi and Hindi	Well	Swimmer
P11	3019	Vietnam	13	Vietnamese	40–49	Woman or female	English and Vietnamese	Very well	Swimmer
P12	3216	India	15	Indian	40–49	Woman or female	English and Hindi	Very well	Non‐swimmer

Results from the interview analysis are presented (*n* = 12) using verbatim quotes from participants. Three key themes emerged regarding how participants determined their swimming ability in different aquatic environments: (1) cultural influences on relationships with waterways and the impact on health literacy, (2) family influence on perceptions of aquatic environments and (3) experience of swimming and water safety education. While each theme is discussed separately, it is important to note they are interrelated and dynamic themes (Table 2).

**TABLE 2:** Key themes and sub‐themes, aligned to literacy theory.

Main theme	Description/inclusions	Literacy theory
Cultural influences in relationship with waterways and the impact on health literacy	Gender Age Culture—perception about swimming and engaging in different aquatic environments. For example, in India, some communities will perform funeral rites by the riverside Regional/rural location (related to access to swimming pools and aquatic environments)	CL Societal and environmental determinants (e.g., culture, language and societal systems)
Family influence on beliefs and practices	Positive and negative associations with water. For example, transgenerational fear and health benefits Social skills reported to shape relationships when determining swimming ability and recreational activities in different aquatic environments	CL Situational determinants (e.g., family and peer influences, physical environment) PL Relationships Motivation
Experience of swimming and water safety education Sub‐themes ○ Experiences in different aquatic locations ○ Application of the education	Age Socio‐economic status Movement skills that allow a person to move on water from one place to another. For example, swimming strokes Self‐perception in how participants describe their swimming proficiency, from swimmers to non‐swimmers Reasons for engaging in movement and physical activity in response to internal or external factors Ability to manage emotions and resulting behaviours in relation to movement and physical activity. For example, overcoming fear to undertake swimming lessons A belief in self‐worth and ability to perform in movement and physical activity. For example, willing to go into deeper water Appreciation and connection to the environment, both built and natural, in relation to movement and physical activity. For example, swimming at different locations or visiting a new beach for a picnic Cognitive factors reported to shape safety and risk, when determining swimming ability and recreational activities in different aquatic environments. For example, the ability/inability to identify rip currents Peer influence on engagement in recreating and swimming in different aquatic environments The influence of the media whether film, TV, radio, news to inform understanding of water safety	CL Personal determinants (e.g., gender, race, socio‐economic status) Situational determinants (e.g., family and peer influences, physical environment) PL Movement skills, moving with equipment, self‐perception, motivation, confidence, connection to place, self‐regulation—emotions, safety and risk

Abbreviations: CL, cultural literacy; HL, health literacy; PL, physical literacy.

### 3.1 Cultural Influences on Relationships With Waterways and the Impact on Health Literacy

Cultural backgrounds and past experiences shaped participants' perceptions of risk and swimming ability in different aquatic environments. For all participants, their relationship with the environments of their birth country influenced their health literacy related to water safety knowledge and perception of swimming ability. For example, beach environments in India and Vietnam were not perceived as places of risk. A few participants were not aware of the dangers at beaches when newly arrived in Australia

When I first came to Australia, my husband was here before me, so he was here one year before I was. And when I went to the beach in Australia, first time I tried to get in water, he told me, “This is not the same as India, you don't go into the water like this over here.” [P12/India/Woman/40‐49/15YIA]

This lower perception of risk in Australia could have been influenced by memories of calmer beaches in India and Vietnam that shaped participants' perceptions of coastal environments.

Isn't it quite interesting that I didn't have an idea to do swimming lesson, even though I'm in a beach city until I go to the river and got the idea. The city that we live in [Vietnam], the beach is, is very peaceful. We never have a high wave or anything. [P11/Vietnam/Woman/40‐49/13YIA]

Furthermore, all participants observed that swimming at the beach was a large part of Australian culture, which was not part of their own experiences living in India and Vietnam. Unlike experiences in their birth countries, learning to swim in Australia was reported as an important skill.

I see less swimming in Indian beaches. Probably because it's not as prevalent in the culture as it is to Australian culture to swim. [P8/India/Man/20‐29/12YIA]

Sociocultural gender norms related to physical activity were observed by two female participants who noted that men and boys were encouraged to learn to swim in India and could be seen frequenting rivers. However, women were discouraged from taking lessons, with one participant noting a stigma that exists in villages, rather than cities, where ‘women don't swim there’ [P7/India/Woman/30‐39/12YIA]. This experience is contrary to that of an Indian female participant whose non‐swimmer father encouraged her and her sister to learn swimming because he believed that ‘survival is important wherever you go’ [P4/India/Woman/40‐49/2YIA].

When reflecting on Australian coastal environments, participants revealed their lack of Australian beach safety knowledge resulted in them almost experiencing a drowning incident.

I nearly died when I went to the [beach name] beach when I was just come to Australia … and it start with a really nice sunny day. [P5/Vietnam/Woman/60‐69/36YIA]

When relating to cultural beliefs, Indian participants reflected on several rivers being holy and respected entities in India, with temples located in close proximity. However, some participants indicated they personally did not practice the same beliefs.

The river is quite spiritual in India … when people die, their ashes are put in the river. [P12/India/Woman/40‐49/15YIA]

Additionally, specific holy rivers could heal ailments.

And I've heard from my friend … she had a very bad skin disease and she visited all doctors … But it wasn't getting cured … Somebody suggested to take a bath in one particular holy river, I can't recollect the name … And that skin disease just wiped. Never came back. [P1/India/Woman/30‐39/7YIA]

Furthermore, for one participant the curative power of water also applied to coastal environments in Goa. This resulted in interactions with water despite not knowing how to swim.

My grandmother, you know, used to believe and a lot of people of all ages used to believe that going and having a saltwater dip … three or four times in the year is very good for your whole body and your system. So, she who didn't know to swim either used to go to the beach. [P6/India/Woman/70‐79/40YIA]

The cultural concept of aquatic environments in India as places of spirituality and healing was also evident in a participant's respect of water besides swimming.

We connect to water … from birth to death. So that's how our life and culture goes. It was not always about swimming. Like water is always life giving. [P4/India/Woman/40‐49/2YIA]

In contrast, a Vietnamese participant reflected on a cultural belief that she personally did not share as a Christian regarding swimming. This negative association related to aquatic environments in Vietnam where fatal drownings had occurred.

They would think that, if you swim at that spot, you might have devil spirit, or that person's spirit pulling your leg, and then you will follow them and then you die. So that's a very common belief, but … it's not my belief. [P5/Vietnam/Woman/60‐69/36YIA]

### 3.2 Family Influence on Perceptions of Aquatic Environments

Participants spoke about how family members such as grandparents and parents influenced their positive and negative associations with aquatic environments, shaping their understanding of water safety and swimming ability in their birth countries. Positive associations included happy memories with family and friends recreating and holidaying by the beach. Regardless of birth country, having picnics, walking along the shore and playing in shallow water or on the sand with family and friends at the beach was a common activity for most participants. In contrast, negative experience included a transgenerational fear of being ‘washed away’ [P9/India/Woman/30‐39/11YIA] by currents and not being able to control the natural environment. Thus, for some participants having parents and grandparents who were fearful of open waterways such as beaches and rivers influenced their own perception of risk and safety in these environments manifesting in behaviour where they would avoid open waterways or deep water. ‘My dad especially was always scared—“Oh, you'll get washed …”—because he didn't know how to swim. And it's such a patriarchal like … man protects the family. And he knows that he can't protect us in water because he doesn't swim. So … I think it was their fear then got passed on to us and we were scared’ [P9/India/Woman/30‐39/11YIA].

Additionally, for another participant, going into deep water was associated with a possibility of suicidal ideation.

My parents … if I say I'm going to go and jump into the water straightaway … or jump into the river, they will think … this person is suicidal or something. [P10/India/Man/30‐39/16YIA]

In contrast, for participants who were learning to swim, having family members who could swim in Australia motivated their participation in adult swimming lessons. For non‐swimmers, responses included ‘missing a lot of fun’ [P7/India/Woman/30‐39/12YIA], to feeling embarrassed about not knowing how to swim in Australia. For a participant with a young child, the impetus to protect the child motivated her undertaking lessons:

If something happens to your child, you can't even go save them. [P7/India/Woman/30‐39/12YIA]

While motivated to learn to swim, the participant found it difficult to practice on her own safely outside of swimming lessons, and did not pursue practicing on her own since.

I try by myself, one day under the water … But that day I don't know what happened. I was not able to push myself up. So, I had to hold the spring next to me to come outside, otherwise I would be dead. [P7/India/Woman/30‐39/12YIA]

However, for some participants, the need to find work to support their families was a priority for them in Australia. This meant that at times the need to learn a new skill such as swimming was a lower priority.

When I come here, like refugee, so I try to go to work … so quite a lot first generation like me in Australia … we just concentrate to work, more than enjoy healthy or something for by yourself. [P2/Vietnam/Man/60‐69/39YIA]

### 3.3 Experience of Swimming and Water Safety Education

Participants' experience of swimming and water safety education influenced their self‐perception of their swimming ability and how they related to different aquatic environments. For nearly all participants, prior to living in Australia, swimming was not perceived as a skill to learn due to the lack of access, availability and affordability of swimming pools and swimming lessons in India and Vietnam. Additionally, for some participants, the drowning experiences of themselves or family members were factors in their awareness and understanding of risk and safety at aquatic environments. Consequently, two sub‐themes emerged: experiences in different aquatic locations and the application of the education.

#### 3.3.1 Experiences in Different Aquatic Locations

For some participants, swimming in India and Vietnam was experienced as a childhood activity with peers without parental supervision in open water environments such as rivers and canals.

They say you have to know how to swim. But they never teach the kids how to swim. The kids just jump to the river and just learn by themselves. In Vietnam, in my era I don't think that they have the swimming class, you know, to teach the kids how to swim. They just swim by themselves. [P3/Vietnam/Woman/50‐59/34YIA]

For nearly all participants, there was a lack of access to swimming pools and formal swimming lessons in their country of birth. Some Indian participants reflected on swimming lessons being expensive and only available privately. Swimming pools were seen as a luxury and could only be found in bigger cities like Mumbai.

None of my friends' swim in outside water. I know only one friend who actually swims. And she used to go to the pool. And it's very expensive in India to access the pool so people don't usually go. [P9/India/Woman/30‐39/11YIA]

For some participants their awareness of safety at Australian beaches became heightened after their non‐fatal drowning experiences or if they had family members who had drowned.

Like before the incident, I wasn't that mindful before my brother's drowning incident … because I never was going into water myself. But I was never mindful about, um, what is rip current? [P1/India/Woman/30‐39/7YIA]

Additionally, participants felt the responsibility of promoting beach safety information as a direct result of their personal non‐fatal drowning experiences. The promotion of this information ranged from community advocacy to informing parents about the meaning of the red and yellow lifeguard flags to signify the presence of lifeguards and a safe swimming zone which are commonplace on Australian beaches, and risks associated with beach environments. For a young male participant, conveying water safety knowledge to his parents was a result of undertaking formal swimming lessons in Australia.

Once we came here, I was the first one to start swimming again … They [parents] kind of got all the swimming knowledge through me. [P8/India/Man/20‐29/12 YIA]

Notably, while some participants felt responsible in promoting beach safety to family and friends, one participant observed that research conducted before visiting a new beach was not focused on water‐related risk.

Like people who I know … we don't really look for what's in the water, whether we go in the water. Normally people look for the … picnic perspective, whether we have toilets near, or barbecue near or whether it's a beautiful beach to take pictures. [P10/India/Man/30‐39/16YIA]

Most participants, when prompted about the use of lifejackets while recreationally boating, said they did wear lifejackets on pedal boats, rowing boats and motorboats. However, a non‐swimmer participant reported that while she always wore a lifejacket, she noted that none of her family members who could swim would wear lifejackets.

#### 3.3.2 Application of the Education

Participants who learned to swim in their birth country did so in swimming pools, rivers, canals, water tanks and home ponds.

I learned from Vietnam, so I can swim for fifty … I just feel, um, embarrassing because it's not look like … the kids they learn from Australia … I swim a bit different with the other technique. [P3/Vietnam/Woman/50‐59/34YIA]

Perception of swimming ability was a key factor in two male participants' confidence in taking risks while swimming at different beach locations that were at times unpatrolled by lifeguards. Despite one male swimmer describing his swimming as ‘raw technique’ [P10/India/Man/30‐39/16YIA], both participants declared they could swim continuously for 50 m in a swimming pool. However, for the older male participant, he admitted to being ‘overconfident’ [P10/India/Man/30‐39/16YIA] in his swimming ability, which he indicated led to his own non‐fatal drowning experience at an unpatrolled Victorian beach after jumping off a pier.

Regarding beach safety knowledge, rip current awareness ranged from having no understanding to being able to identify rip currents via visual images. Notably, two participants who identified as swimmers and had undertaken formal swimming lessons in Australia experienced difficulty in identifying rip currents.

I was taught how to look out for them, but I don't remember. [P8/India/Man/20‐29/12YIA]

Additionally, both participants, when probed, were not aware of the Surf Life Saving BeachSafe app as a resource. The female participant admitted to only swimming in swimming pools due to the presence of lifeguards. One participant recommended beach safety warnings could include promotion of the number of drownings at that location and whether it was safe to walk or wade in the water.

People have been just wading in the water, not swimming, just wading, walking in the water … very close to sand. But they got pulled away by the rip currents. [P1/India/Woman/30‐39/7YIA]

When participants reflected on their understanding of the Australian red and yellow lifeguard flags and general beach safety, responses ranged from understanding what the flags were, to stating they had never seen such a flag before.

Some participants indicated flags were not markers of safe areas to swim in India or Vietnam and were used for signifying hazards.

… in Vietnam, when the water is deep, or the wave is too strong, they going to put … like a black flag right in that area. So, whenever we see that flag, we're not going to swim around that area. [P11/Vietnam/Woman/40‐49/13YIA]

Other participants expressed confusion about the purpose of the flags and made recommendations to make the information clearer.

So, the message about saying that please “swim between the flags”—if the flags or if the lifeguards are not there, what are the tourists supposed to do? Are they even supposed to go in the water or not? [P1/India/Woman/30‐39/7YIA].

If you actually have an image of a flag—and then two flags and a person swimming in the middle with a tick on top, then I'm like, okay, so that's what the flag looks like, and that's where I'm allowed to swim. [P9/India/Woman/30‐39/11YIA].

## 4 Discussion

This study explored how Indian and Vietnamese communities in Victoria determined their health literacy and applied this knowledge in different aquatic environments. For all, living in Australia was associated with a deep appreciation, enjoyment and desire for swimming as a leisure activity and survival skill. However, for some, prior to living in Australia, swimming was not perceived as a skill to learn due to lack of access, availability and affordability of swimming pools and swimming lessons in India and Vietnam, and due to family and homeland cultural influences. Childhood experiences and family influences of migrants' homelands are influential on their sport and leisure practices [27]. Hulteen et al. propose viewing participation in leisure‐time physical activities through a filter where sociocultural aspects and geographic location are influential factors in determining the importance of the type of physical activity undertaken [28]. Thus, when viewed through these filters, participants' perception of swimming changed, acknowledging the importance of learning the skill in Australia.

In factoring cultural literacy as one of the domains of health literacy, it emerged for some of the Indian female participants that gender was a barrier in accessing swimming lessons in India, where men and boys dominated aquatic environments. In Australia, gender roles for CALD women have been reported as a barrier to physical activity [16]. For two male participants, their perceived swimming ability gave them confidence in swimming at different beach locations in Australia that were at times unpatrolled by lifeguards. This research found that confidence and self‐perception of swimming ability likely played a role in males taking more unperceived risks in different waterways. In Victoria, 76% of CALD people who fatally drowned between 2012 and 2022 were male, where incidents typically occurred in open waterways [2].

Additionally, both men in the current study also undertook the role of intercultural mediators advising parents of beach safety, as they were able to immerse themselves in Australian culture through interaction with peers and access to education [29]. A recommendation arising from this finding would be for water safety education targeting Asian adults to promote water safety knowledge (such as knowledge of red and yellow lifeguard flags in the Australian context and rip currents in the beach context, and wearing of lifejackets when fishing and boating) to their family and parents, in consideration of the importance of family values in Indian and Vietnamese culture. This recommendation is also reflected in the *Aquatic Injury Prevention Agenda 2024–2025* advocacy recommendation to encourage and empower CALD communities to educate their families, friends and communities about water safety. However, it is critical that the person who takes on this role understands the water safety risks and is competent themselves.

This study found vast differences in beach safety knowledge between participants. With the Australian beach safety message ‘swim between the flags’ [30], responses ranged from understanding the message to never seeing a lifeguard flag. Participants reported flags not being markers of safety in India or Vietnam. For one Vietnamese participant, flags at a beach signified a hazard. Similarly, in a study exploring Japanese university students' beach safety knowledge in Australia, 60% of students interpreted the red and yellow flags as signifying a danger zone [31]. Our recommendation supports a review of the promotion of the red and yellow lifeguard flags to incorporate more universal visual symbols to indicate safe and unsafe swimming zones, noting that it is impractical to display safety messages in multiple languages on static signages. Future drowning prevention work should consider multisectoral policies and partnerships across education providers, health and migration sectors [32] to promote awareness of the Surf Life Saving BeachSafe App [33], multicultural swimming programmes [34], the significance of the red and yellow flags, the dangers of rip currents and the high rates of drowning deaths during summer. Innovative health promotion campaigns targeted towards multicultural communities to promote water safety knowledge could be adopted, similar to the success seen elsewhere such as the ‘Drama Downunder’ campaign which targeted Australian gay men to increase sexual health‐seeking behaviour. This campaign relied on outdoor advertisements, including billboards, train stations, and tram stops, as well as promotion via gay media, social events and venues, with information provided by a campaign website and free short message service (SMS) reminders [35]. Such an approach could be applied to a multicultural water safety campaign, with memorable images, messages and widespread promotion across various advertisements and relevant multicultural media channels to increase water safety knowledge.

This current research also identified that some participants who had exposure to water safety education were able to identify rip currents in photographs, contrasting previous studies of Asian participants who lacked rip current awareness [8, 31]. Between 2023 and 2024, rip currents contributed to one in three Australian beach drowning deaths [36], with many incidents resulting from a lack of awareness of rip currents and how to escape them. Accordingly, future drowning prevention strategies should consider providing education on rip currents to CALD communities, including the risks posed when walking or wading in the water. Our recommendation is to encourage a knowledge transfer of water safety skills into different aquatic environments [37], by developing a programme that incorporates physical literacy learning development over a person's life course. This could involve co‐designing programmes with Indian and Vietnamese community leaders and bicultural workers [38] that involve a whole‐of‐family approach (social) where family members are encouraged to participate in swimming (physical) and water safety training (cognitive) by building confidence (psychological) in deep water at beaches and rivers. Importantly, multilingual water safety programming in environments familiar to participants has been found to be key to the acceptance and adoption of water safety behaviour, thus programmes should consider these factors in design and delivery [39].

### 4.1 Strengths and Limitations

This study was strengthened by the sample population drawn from under‐served populations—Indian and Vietnamese communities in Victoria. Another strength was the lead researcher's experience as a swim teacher and having learnt to swim as an adult migrant. As a research team, it was decided not to disclose this swim teacher experience to reduce social desirability bias. In contrast, from an insider or emic perspective, the lead researcher's experience as a migrant adult swimmer could have inspired a sense of trust with participants [40]. Furthermore, the accuracy and integrity of the research process involved respondent validation or member checking where participants were invited to review their interview transcripts [24].

A limitation was the possibility that some participants who were attracted to this research had themselves experienced an aquatic‐related incident or had family members who had fatally drowned, which may not reflect perspectives from people from Indian and Vietnamese communities who have not had these extreme experiences [41]. Furthermore, the heterogeneous sample in terms of the uneven distribution of participants across countries and genders (four Vietnamese people and three men) means that we were unlikely to have achieved data saturation in terms of perspectives from men and Vietnamese people particularly. Another limitation was the focus on individuals who could communicate in English and provide informed consent. Although considering fatal drownings in Victoria have occurred among CALD individuals who had lived in Australia for an average of 20 years [2], we believed it was still relevant to recruit from English speakers. Nevertheless, the novelty of this research and the insights obtained from the qualitative approach contribute to the growing body of literature related to drowning prevention in adult migrants in Australia [32, 42, 43].

## 5 Conclusion

Drowning continues to be a public health issue in Australia, especially for CALD communities. This study highlighted that cultural experiences and practices in aquatic settings, family influences on engagement and interaction with waterways and homeland experiences continued to influence Indian and Vietnamese participants' perception of their swimming ability and understanding of risk and safety in different aquatic environments. Current drowning prevention practices and strategies in Australia, such as the ‘swim between the flags’ message may not be applicable or successfully translated to these communities due to their unfamiliarity with what flags signify, the lack of clear translation of the meaning of ‘swim’ and their misunderstanding of risk at unpatrolled waterways. To consider cultural influences in relationships with waterways and the impact on health literacy when promoting and educating on water safety to Indian and Vietnamese communities, drowning prevention initiatives could be co‐designed with Indian and Vietnamese community leaders. Such action is required urgently due to the increasing representation of Indian and Vietnamese populations within Victorian drowning statistics.

## Funding

Funding to the maximum value of $300 was obtained from Deakin University's School of Health and Social Development Honours Support Programme to support this research.

## Ethics Statement

This study has received Deakin University ethics approval (reference number: 2024‐079).

## Conflicts of Interest

H.G. works full‐time at Life Saving Victoria and S.W.‐P. works full‐time at Royal Life Saving Society—Australia. The other authors declare no conflicts of interest.

## Supporting information


**Table S1:** Codebook framework.

**Table S2:** CORE Q (Consolidated Criteria for Reporting Qualitative Research)—32 Item checklist for reporting qualitative research [19].

**Table S3:** Interview questions and guiding theory.

**Table S4:** Interview questions with example visual prompts.

## Data Availability

The data that support the findings of this study are available on request from the corresponding author. The data are not publicly available due to privacy or ethical restrictions.
