# Supplementary material for: Exploring the Swimming and Water Safety Behaviour Among Indian and Vietnamese Adults in Australia
Source: Health Promot J Austr. 2026 Mar 2;37(2):e70163. doi: 10.1002/hpja.70163 (PMC12953056; doi:10.1002/hpja.70163)
Supplement: Supplementary file 1 — Table S1: Codebook framework. [file HPJA-37-0-s004.docx]

## Supplementary Table 1 - Codebook Framework

| **Category** |  | **Code** |  | **Definition** | **Notes** |
| --- | --- | --- | --- | --- | --- |
| Personal determinant (eg age, gender, race, socio economic status, education, occupation, employment, income, literacy) |  | 1. Gender |  | Gender framing lens. Person describes event related to gender. | For eg, interviewee observes only men and boys swimming in Indian rivers. Women not encouraged to undertake swimming |
|  |  | 1. Age |  | Age framing lens. Person describes event related to age. For eg. this can relate to the age a person learns to swim, where the person learns and how they learn. |  |
|  |  | 1. Socio economic status |  | The description of socio economic status including status and privilege as exclusive markers to denote wealth and ease of access to resources (for eg swimming lessons)  This also includes low socio economic status & barriers to access  Include employment | Houses along the beachfront at Chennai denote wealth and privilege, this is true of most beachfront residences. |
|  | Sub-component of Personal Determinant:  **Physical Literacy** | **Physical**  Physical factors reported to shape movement skills when determining swimming ability in different aquatic environments | 1. Element: movement | Movement skills that allow a person to move on water from one place to another.  This could relate to  •Swimming strokes, gliding  •Floating, diving  This could range from learning to swim, to performing proficient survival and rescue techniques in a pool. |  |
|  |  | **Psychological**  Psychological factors reported to shape connection to place, confidence, self-perception, motivation, managing emotional responses when determining swimming ability & recreational activities in different aquatic environments | 1. Element: Self-perception | Understands self in relation to movement and physical activity and recognises personal strengths and areas for development. This can include how participants describe their swimming proficiency, whether by distance they can swim, or by the type of swimming they can do. This definition also includes participants who would describe themselves as non-swimmers. | Out of all participants, two participants have had external observers regarding their swimming ability |
|  |  |  | 1. Element: Motivation | Reasons for engaging in movement and physical activity in response to internal or external factors.  For eg • Participant’s family members can swim• Participating in physical activity for enjoyment with family and friends | Eg Oldest participant discloses now she has the time to take up swimming lessons after supporting her children. |
|  |  |  | 1. Element: Self-regulation (Emotions) | Ability to manage emotions and resulting behaviours in relation to movement and physical activity. This includes acknowledging a personal fear in encountering different aquatic environments, however at this stage, the attitudes and emotions haven’t been developed as yet. | What it could look like:  • Channeling frustration into motivation to take up swimming lessons  • Overcoming nervousness to undertake swimming lessons  • Regulating/overcoming fear of water  • For participants who can’t swim, would be vocal about their fear of water.  Fear of open water, whether the beach or river. Inability to have control in the water creates fear, at the mercy of surroundings |
|  |  |  | 1. Element: Confidence | A belief in self-worth and ability to perform in movement and physical activity.  What it could look like:  •Swimming at new locations  •Willing to go into deeper water | One participant built confidence living close to beaches in Qld, and transferred this confidence to beaches in Victoria |
|  |  |  | 1. Element: Connection to place | Appreciation and connection to the environment, both built and natural, in relation to movement and physical activity.  What it could look like:  •Seeking different, yet familiar environments to do a preferred swim/recreation activity e.g. swimming at different locations or visiting a new beach for a picnic  •Participating in different swim/ recreation activities because of a connection to that environment e.g. swimming in a local river |  |
|  |  | **Cognitive**  Cognitive factors reported to shape safety & risk, when determining swimming ability & recreational activities in different aquatic environments | 1. Element: Safety & risk | The range of this element includes being unaware of water safety information to having the potential to develop understanding of risks, risk-management and safety considerations for self and others in different aquatic environments.  This can include knowing how to call for help when in trouble, knowing where to access water safety information for eg BeachSafe app and Beach safety signs | What it could look like:  • Unable to identify rip currents  • Unable to identify water safety information  • Understanding and obeying safety rules and procedures at the beach/ swimming pool   - Perceived danger such as crocodiles in the river   • Warning friends and family about the risk of currents  Example: Being aware/ unaware of the red and yellow flags, where to swim.  Assessing risk (observing others)  Participants would note if there were people on the beach to articulate safety |
|  | Sub-component of Personal Determinant:  **Physical Literacy** | **Social**  Social skills reported to shape relationships when determining swimming ability & recreational activities in different aquatic environments | Element: Relationships | Building and maintaining respectful relationships that enable a person to interact effectively with others. | What it could look like:  • Showing awareness for the feelings, needs and interests of others, for eg appreciate that Australian husband grew up swimming and loves the water |
| Societal and environmental determinants (eg demographic situation, culture, language, political forces, societal systems) |  | 1. Culture | 1. Beliefs | Perception about swimming and engaging in different aquatic environments via a cultural framing lens. Person describes event related to cultural background. A sub theme to this is ‘Beliefs’ which relates specifically to specific beliefs related to the participant’s culture | This could include for example, jumping into deep water indicates suicidality  In India, some communities will perform funeral rites by the riverside.  Adult supervision? |
|  |  | 1. Regional |  | Regional/rural location, away from metropolitan/urban environment | This code could relate to access to swimming pools. Also there was an example where a participant observed that those who lived in rural areas in India knew how to swim vs those in urban areas |
| Situational determinants (e.g. social  support, family and peer influences, media use and physical  environment). |  | 1. Family influence |  | Family influence on engagement in recreating and swimming in different aquatic environments.  This can also include positive and negative associations with water for eg transgenerational fear and health benefits | Swimming within the family is not perceived as important. Individual explains that no one in the family can swim, this can include parents, siblings and grand parents  Grandmother says to participant when she says she will take swimming classes, ‘Oh no. Be careful, because if you're in the water for too long, you can get cramps and then you’ll drown.’  Parents have particular fear of swimming and deep water |
|  |  | 1. Peer influence |  | Peer influence on engagement in recreating and swimming in different aquatic environments | Childhood memories of learning to swim by the river with friends, but not parents. Peer influence often cited where parents are too busy to supervise. |
|  |  | 1. Mass media influence |  | The influence of the media whether film, TV, radio, news to inform understanding of water safety |  |
