# Supplementary material for: Exploring the Swimming and Water Safety Behaviour Among Indian and Vietnamese Adults in Australia
Source: Health Promot J Austr. 2026 Mar 2;37(2):e70163. doi: 10.1002/hpja.70163 (PMC12953056; doi:10.1002/hpja.70163)
Supplement: Supplementary file 2 — Table S2: CORE Q (Consolidated Criteria for Reporting Qualitative Research)—32 Item checklist for reporting qualitative research [19]. [file HPJA-37-0-s003.docx]

**Supplementary Table 2: CORE Q (Consolidated Criteria for Reporting Qualitative Research) – 32 Item checklist for reporting qualitative research (**Tong, Sainsbury & Craig, 2007).

| **Item** | **Topic/Domain** | **Guide questions/description** | **Where in the manuscript** | **Included** |
| --- | --- | --- | --- | --- |
| **Domain: Research team & reflexivity** | | | | |
| ***Personal Characteristics*** | | | | |
| 1 | Interviewer/facilitator | Which author/s conducted the interview or focus group? | Methods: Research team and reflexivity | Yes |
| 2 | Credentials | What were the researcher’s credentials? | Methods: Research team and reflexivity | Yes |
| 3 | Occupation | What was their occupation at the time of the study? | Methods: Research team and reflexivity | Yes |
| 4 | Gender | Was the researcher male or female? | Methods: Research team and reflexivity | Yes |
| 5 | Experience and training | What experience or training did the researcher have? | Methods: Research team and reflexivity | Yes |
| ***Relationship with participants*** | | | | |
| 6 | Relationship established | Was a relationship established prior to study commencement? |  | No |
| 7 | Participant knowledge of the interviewer | What did the participants know about the researcher? | Methods: Research team and reflexivity  Participants gained knowledge about the research project as part of the researcher's role as a Public Health and Health Promotion Honours student via the Plain Language Statements as part of the informed consent process. | Yes |
| 8 | Interviewer characteristics | What characteristics were reported about the interviewer/facilitator? | Methods: Research team and reflexivity  Discussion: Strengths and limitations | Yes |
| **Domain: study design** | | | | |
| ***Theoretical framework*** | | | | |
| 9 | Methodological orientation and Theory | What methodological orientation was stated to underpin the study? | Methods: Design and Materials | Yes |
| ***Participant selection*** | | | | |
| 10 | Sampling | How were participants selected? e.g. purposive, convenience, consecutive, snowball | Methods: Participants and recruitment | Yes |
| 11 | Method of approach | How were participants approached? | Methods: Participants and recruitment | Yes |
| 12 | Sample size | How many participants were in the study? | Results: paragraph 1 | Yes |
| 13 | Non-participation | How many people refused to participate or dropped out? Reasons? | Methods: Research team and reflexivity  Participants were given the options to opt-out of the research if they chose. | Yes |
| ***Setting*** | | | | |
| 14 | Setting of data collection | Where was the data collected? e.g. home, clinic, workplace | Methods: Data collection | Yes |
| 15 | Presence of non-participants | Was anyone else present besides the participants and researchers? | Methods: Data collection | Yes |
| 16 | Description of sample | What are the important characteristics of the sample? | Results: Paragraph 1 and Table 1 Participant Demographics | Yes |
| ***Data Collection*** | | | | |
| 17 | Interview guide | Were questions, prompts, guides provided by the authors? Was it pilot tested? | Methods: Materials, Data collection and supplementary tables 3 & 4 | Yes |
| 18 | Repeat interviews | Were repeat interviews carried out? If yes, how many? | NA | NA |
| 19 | Audio/visual recording | Did the research use audio or visual recording to collect the data? | Methods: Data collection | Yes |
| 20 | Field notes | Were field notes made during and/or after the interview or focus group? | Methods: Analysis | Yes |
| 21 | Duration | What was the duration of the interviews or focus group? | Methods: Data collection | Yes |
| 22 | Data saturation | Was data saturation discussed? | Discussion: Strengths and Limitations | Yes |
| 23 | Transcripts returned | Were transcripts returned to participants for comment and/or correction? | Methods: Analysis  Discussion: Strengths and Limitations | Yes |
| **Domain: analysis & findings** | | | | |
| ***Data analysis*** | | | | |
| 24 | Number of data coders | How many data coders coded the data? | Data collection: Analysis | Yes |
| 25 | Description of the coding tree | Did authors provide a description of the coding tree? | Supplementary Table 1 | Yes |
| 26 | Derivation of themes | Were themes identified in advance or derived from the data? | Methods: Analysis and Results: Paragraph 1 | Yes |
| 27 | Software | What software, if applicable, was used to manage the data? | Data collection and Analysis | Yes |
| 28 | Participant checking | Did participants provide feedback on the findings? | Methods: Analysis | Yes |
| ***Reporting*** | |  |  |  |
| 29 | Quotations presented | Were participant quotations presented to illustrate the themes / findings? Was each quotation identified? e.g. participant number | Results – throughout and Table 2 | Yes |
| 30 | Data and findings consistent | Was there consistency between the data presented and the findings? | Discussion | Yes |
| 31 | Clarity of major themes | Were major themes clearly presented in the findings? | Results and Table 2 | Yes |
| 32 | Clarity of minor themes | Is there a description of diverse cases or discussion of minor themes? | Results and Table 2 - key themes and sub-themes aligned to literacy theory presented  Discussion – major themes and selected sub-themes discussed in context of the wider study | Yes |
