# Supplementary material for: Exploring the Swimming and Water Safety Behaviour Among Indian and Vietnamese Adults in Australia
Source: Health Promot J Austr. 2026 Mar 2;37(2):e70163. doi: 10.1002/hpja.70163 (PMC12953056; doi:10.1002/hpja.70163)
Supplement: Supplementary file 3 — Table S3: Interview questions and guiding theory. [file HPJA-37-0-s002.docx]

## Supplementary Table 3 - Interview questions and guiding theory

| **Main question** | **Prompting questions** | **Literacy theory** |
| --- | --- | --- |
| 1. How often do you go to the beach, river or swimming pool? | What do you do when you visit? (Prompt: swimming/fishing/paddle-board/boating, walking at the beach or river. If no activity, have you ever wanted to? And why haven’t you?) | HL (Access)  PL (Movement skills, Moving with equipment, Self-perception, Motivation, Confidence, Connection to place, Safety and risk) |
| 1. What were your earliest memories of playing or spending time around the beach, river or swimming pool? | Was this with your parents or friends? Was this in Australia or elsewhere? | CL (Situational and Personal determinants)  PL (Connection to place, Society and Culture) |
| 1. Have you received advice from friends, family and/or community members about swimming, fishing or playing near the water? | What are the messages? | HL (Access/Understand/Appraise/Apply)  CL (Situational determinants) |
| 1. Could you describe any stories or beliefs about swimming or being at the beach or river? |  | CL (Societal & environmental and situational determinants)  PL (Connection to place, Society & Culture) |
| 1. Have you done swimming lessons? If yes, who taught you?   How easy was it to access lessons? | Prompt: parents, family members, at school, private lessons  Prompt: If you haven’t taken lessons, would you consider taking lessons? Why/why not? | HL (Access)  PL (Movement skills, (Connection to place, Society and Culture)  CL (Personal/Situational/ Societal & environmental determinants) |
| 1. Do you think you are a good swimmer? | Prompt: is it from experiences, education, other people? Why do you think you’re a good swimmer? Are there any other reasons? | PL (Movement skills, Self-perception) |
| 1. How far can you swim in the pool? What sort of swimming? For e.g. freestyle, backstroke, snorkelling, diving, treading water? | Prompt: VWC Safety certificate competencies:  -Could you swim continuously for 50metres? (2 laps in a 25m pool)  -Surface dive and swim underwater to search and recover an object  -Scull, float and tread water for 2 minutes  -Swim survival strokes for 3 mins  - Respond to an emergency by demonstrating DRSAB and the recovery position.  - Perform rescue skills | PL (Movement skills) |
| 1. Before visiting a new beach or river, do you plan how to be safe in case you get into trouble? | Prompt: what would you do in an emergency? | HL (Access/ Understand/Appraise/Apply)  PL (Cognitive/Psychological) |
| 1. Have you used any apps or websites before deciding to visit a beach or river? | Prompt: For example, the BOM website or SLS Beach Safe app. Why/why not?) | HL (Access/Understand/ Appraise/Apply) |
| 1. Have you experienced accidentally getting into trouble while swimming, or seen someone getting into trouble? | Prompt: How do you get help if you got into trouble? | HL (Understand/Appraise/Apply)  PL (Knowledge and risk) |

HL = Health Literacy, PL = Physical Literacy, CL = Cultural Literacy
