# Supplementary material for: Exploring the Swimming and Water Safety Behaviour Among Indian and Vietnamese Adults in Australia
Source: Health Promot J Austr. 2026 Mar 2;37(2):e70163. doi: 10.1002/hpja.70163 (PMC12953056; doi:10.1002/hpja.70163)
Supplement: Supplementary file 4 — Table S4: Interview questions with example visual prompts. [file HPJA-37-0-s001.docx]

## Supplementary Table 4 - Interview questions with example visual prompts

| **Main question** | **Image** | **Literacy Theory** |
| --- | --- | --- |
| 1. When you visit a new beach, do you usually read the public beach safety signage? Do you think the signage is effective? | Figure 1: Beach safety sign at Altona Beach. (Photo: Lian Low)  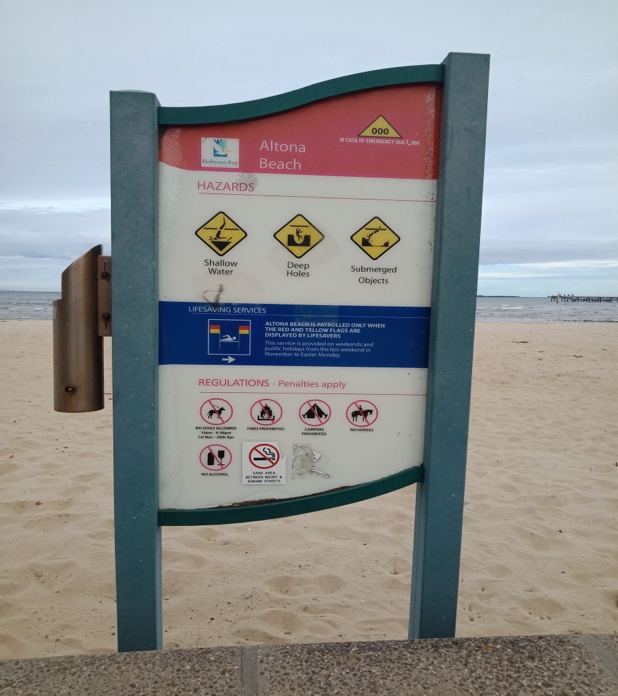 | HL (Access/  Understand/  Appraise/  Apply)  PL (Knowledge and risk) |
| 1. When you go to the beach, what do you notice is the difference to how people go to the beach here in Australia? (Prompt: Picnic/Walking/Swimming) | 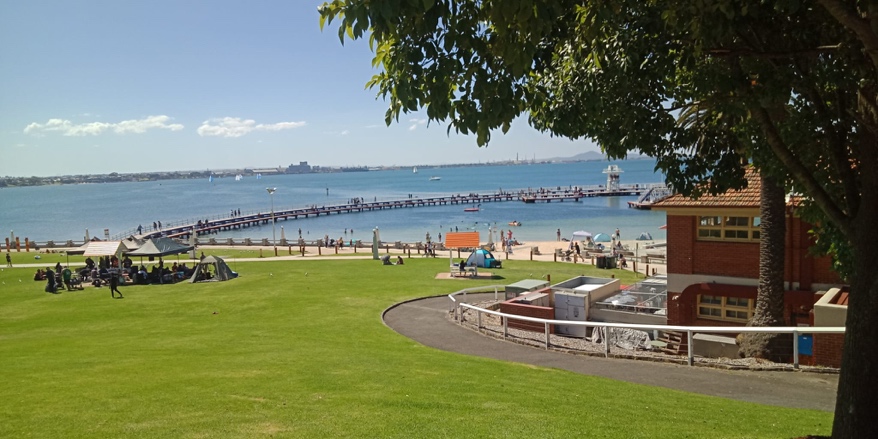Figure 2: Eastern Beach, Geelong, Victoria (Photo: Lian Low) | CL (Societal & environmental and situational determinants)  PL (Movement skills, (Connection to place, Society and Culture) |
| 1. In this river scene in China, are there resonances with how you relate to rivers in your birth country? (Prompt: Swim clothes) | 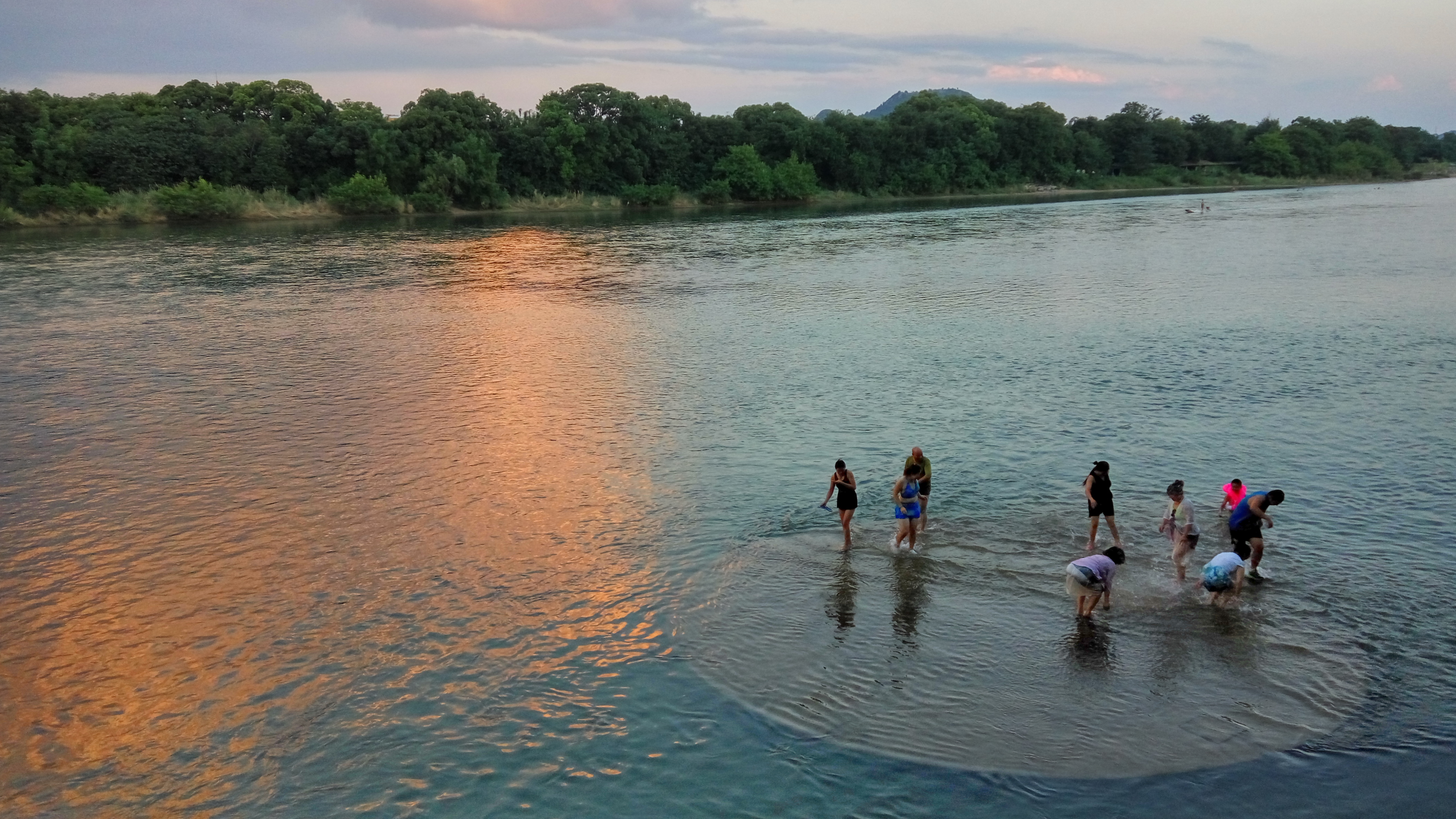  Figure 3:  ["Locals playing in the Guilin section of the Li River in Guanxi Province, China"](https://en.m.wikipedia.org/wiki/File:Li_River.jpg) is licensed under [CC BY-SA 4.0](http://creativecommons.org/licenses/by-sa/4.0) | CL (Societal & environmental and situational determinants)  PL (Movement skills, (Connection to place, Society and Culture) |
| 1. Have you visited any rivers in regional Australia? (Prompt: Murray River. Would you consider swimming? Have you gone fishing or boating? If you do, would you wear a life jacket?) | 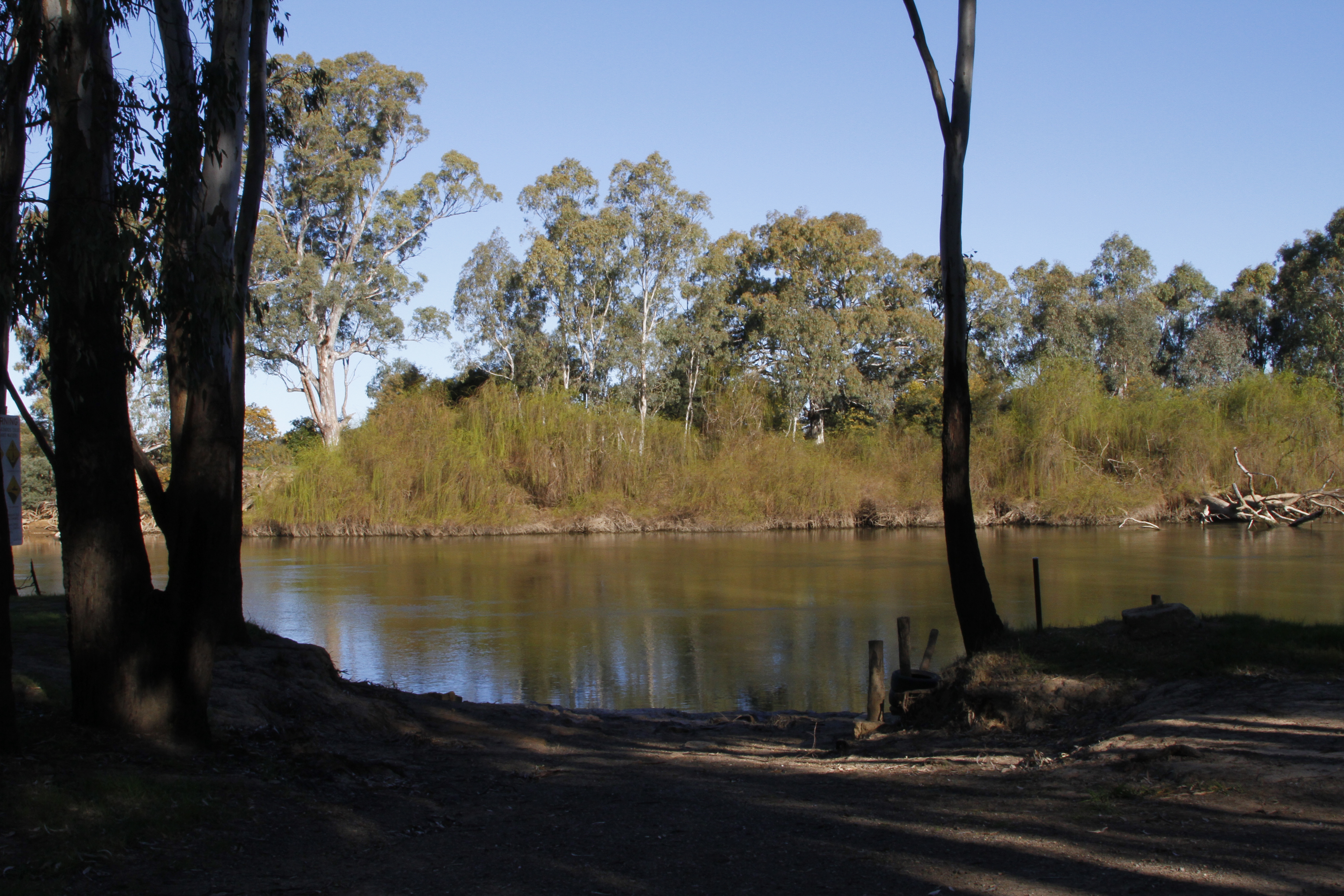  Figure 4 "[The mighty Murray river - The view from the house](https://www.flickr.com/photos/90851177@N00/15181470458)" by [Percita](https://www.flickr.com/photos/90851177@N00) is licensed under [CC BY-SA 2.0](https://creativecommons.org/licenses/by-sa/2.0/?ref=openverse). | PL (Movement skills & Moving with equipment, Connection to place, Society and Culture)  HL (Access/Understand/  Appraise/  Apply) |
| 1. In the last 3 photographs, can you identify where is it safe to swim?   (Prompt: Can you tell me what a rip current is) | 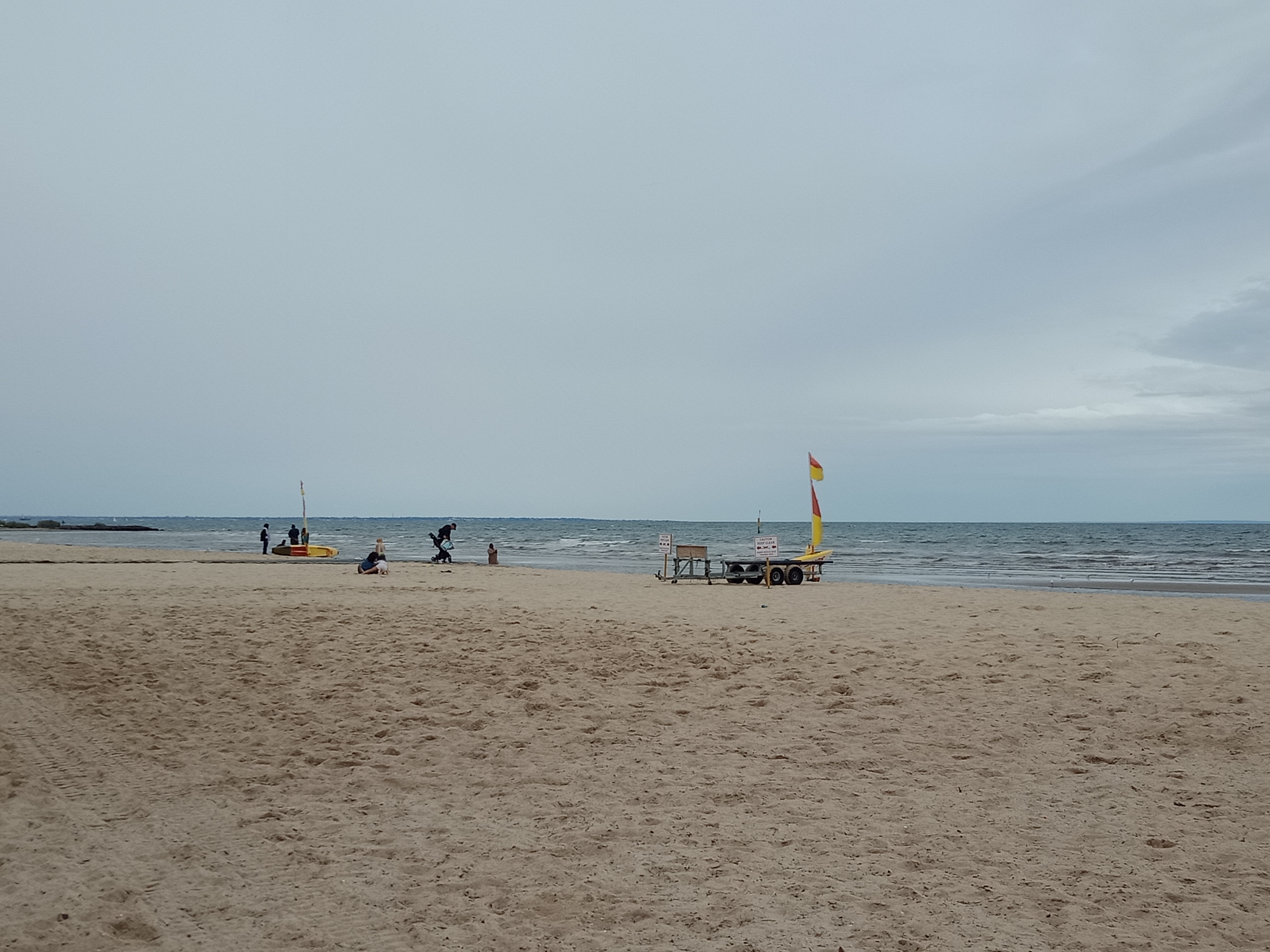  Figure 5 Altona Beach with lifeguard red and yellow safety flags. (Photo: Lian Low)  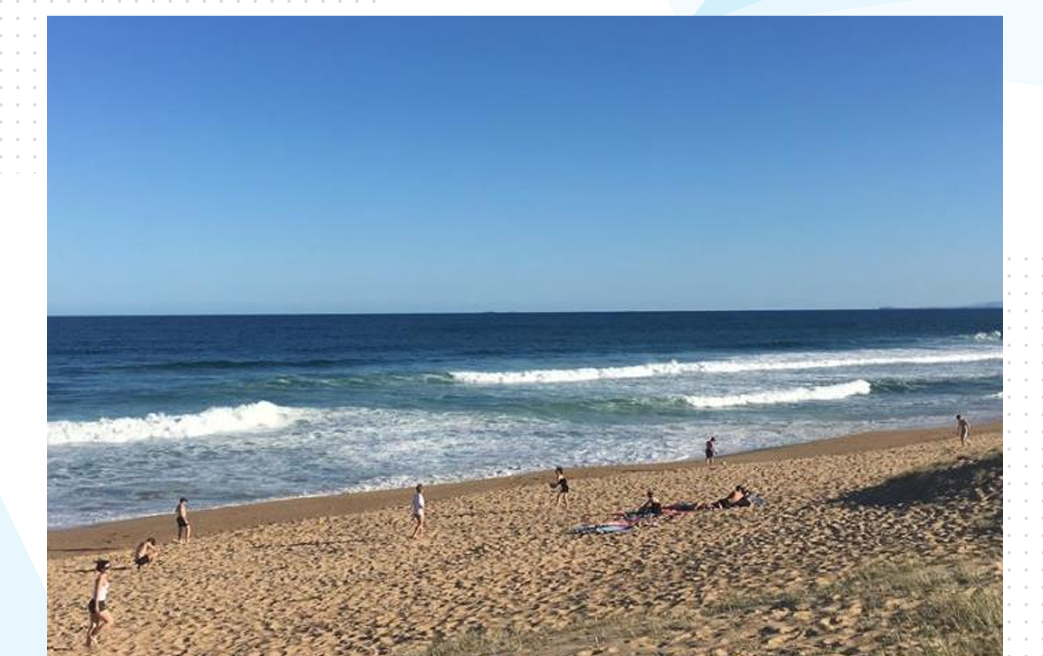  Figure 6 October 2017, Stanwell Park, NSW, photo courtesy of Prof Rob Brander, UNSW.  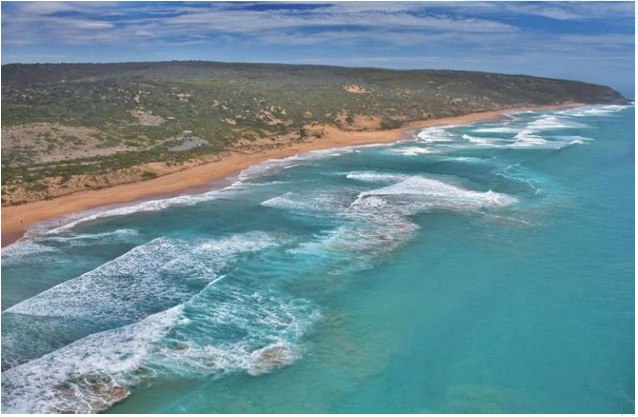  Figure 7 February 2017, Watipinga Beach, SA,  photo courtesy of Shane Daw, Surf Life Saving Australia. | HL (Access/ Understand/  Appraise/  Apply)  PL (Movement skills & Moving with equipment, Connection to place, Society and Culture) |

Abbreviations: HL, Health Literacy; CL, Cultural Literacy; PL, Physical Literacy
